# Bacteria and Sepsis: Microbiome to the Rescue?

**DOI:** 10.3390/jcm10163578

**Published:** 2021-08-14

**Authors:** Hansol Kang, Ryan M. Thomas

**Affiliations:** 1 University of Florida College of Medicine, Gainesville, FL 32610, USA; hansolkang@ufl.edu; 2 Department of Surgery, University of Florida College of Medicine, Gainesville, FL 32610, USA; 3 Department of Molecular Genetics and Microbiology, University of Florida College of Medicine, Gainesville, FL 32610, USA; 4 North Florida/South Georgia Veterans Heath System, Gainesville, FL 32608, USA

**Keywords:** microbiome, microbiota, sepsis, infection, prebiotic

## Abstract

The microbiome is the metagenome of all microbes that live on and within every individual, and evidence for its role in the pathogenesis of a variety of diseases has been increasing over the past several decades. While there are various causes of sepsis, defined as the abnormal host response to infection, the host microbiome may provide a unifying explanation for discrepancies that are seen in septic patient survival based on age, sex, and other confounding factors. As has been the case for other human diseases, evidence exists for the microbiome to control patient outcomes after sepsis. In this review, associative data for the microbiome and sepsis survival are presented with causative mechanisms that may be at play. Finally, clinical trials to manipulate the microbiome in order to improve patient outcomes after sepsis are presented as well as areas of potential future research in order to aid in the clinical treatment of these patients.

## 1. Introduction

It is estimated that ≈1.7 million patients in the United States annually will succumb to an episode of sepsis, which is defined as life-threatening organ dysfunction caused by the dysregulated host response to infection [1]. The care of these patients results in a significant financial burden on the national healthcare system, with approximately 6.2% of the annual healthcare expenditure of the United States devoted to the care of these patients [2]. Additionally, given the median age of sepsis diagnosis of 67 years [3], there is a significant number of individuals who die or are unable to return to the workforce secondary to a septic episode and its long-term complications, leading to significant costs to society [4]. While advances in the critical care of these patients has resulted in improved survival, there is still a large portion of patients, particularly the elderly, who consistently have worse outcomes [5,6,7]. Thus, it is imperative to identify factors that influence these observations.

The microbiome is the collection of microbes and their associated metagenome, consisting of bacteria, viruses, fungi, archaea, and protozoa that create a symbiotic, pathobiont, or commensal relationship with its host, whereas the microbiota refers to the specific microbial species that create the community structure. Research into the role of the microbiome in a variety of disease processes has revealed their critical role in health and illness [8,9], and its potential role in modulating outcomes of patients with sepsis is starting to be recognized [10]. Given the current plateau in treatment options for patients with sepsis, the identification or modulation of host factors at the time of a septic insult may identify patients at high risk for poorer outcomes and provide a needed clinical advantage to treat patients. Herein, we highlight recent research that supports the role of the microbiome in sepsis and septic complications, and outstanding questions will be addressed to take advantage of the host microbiome to improve patient outcomes.

## 2. The Microbiome in Sepsis

### 2.1. Background and Associations

**Figure 1:**
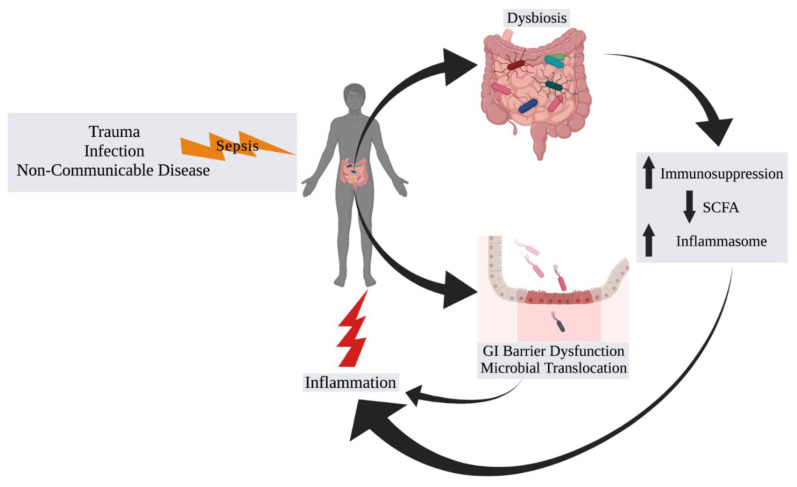
Interactions of the Microbiome with Sepsis. An inciting event of sepsis such as trauma, infection, or complications from a non-communicable disease result in an alteration in the normal homeostatic state of the microbiome (“dysbiosis”), which leads to increased immunosuppression and inflammasome assembly and activation, and/or decreased short-chain fatty acid (SCFA) production by gut microbiota. The resulting effect is increased and persistent systemic inflammation. Additionally, the septic insult can result in gastrointestinal (GI) barrier function with microbial translocation and systemic inflammation.

The etiology of sepsis is variable and can be caused by infections, injury/trauma, and non-communicable diseases with gastrointestinal and pulmonary diseases responsible for a vast majority of cases worldwide over the past 30 years [11]. The nidus for infectious causes may further be divided between community-based or nosocomial. Regardless of the etiology, the downstream physiologic responses and perturbations are similar. The human immune system has evolved to recognize damage-associated molecular patterns (DAMPs) and pathogen-associated molecular patterns (PAMPs) [10]. Damage to host cells results in the release of cytosolic, nuclear, and mitochondrial proteins and metabolites [12,13]. These subsequently bind to a variety of pattern recognition receptors (PRRs) such as the family of Toll-like receptors (TLRs). These TLRs have a significant role in innate immune modulation. For example, TLRs propagate signal transduction pathways that modulate expressions of genes involved in the production of cytokines, chemokines, and type I interferons, which orchestrate inflammation [14]. The PAMPs are conserved motifs derived from microbes that likewise bind to the TLR family of PRRs. Prototypical PAMPs are bacterial lipopolysaccharide (LPS, derived from the cell wall of Gram-negative bacteria), flagellin, and lipoteichoic acid (from the cell wall of Gram-positive bacteria), which bind to TLR4, TLR5, and TLR2, respectively. Activation of the TLR pathways results in the upregulation of the innate immune system, particularly macrophages, dendritic cells, and natural killer (NK) cells [15]. The activation of these cells results in a multifactorial pro- and anti-inflammatory immune pathway response [16,17]. Although this balance is typically finely tuned, often, the processes can become dysregulated, resulting in uncontrolled and persistent pro-inflammatory or immunosuppressive pathway activation from which a patient may never recover [18,19]. Thus, identifying factors that result in this uncontrolled or persistent immune imbalance is critical to improve patient outcomes. Several factors have been associated with differences in sepsis survival. Most have been reported differences in sex [20,21,22,23], age [5,7,23,24], and race [22,23,25,26]. For example, men are reported to have higher incidence of sepsis compared to women with a mean annual relative risk of 1.28 (95% confidence interval, 1.24–1.35) [20,21,23]. Furthermore, older individuals tend to have worse survival than younger, with an odds ratio for mortality of 2.26 (95% confidence interval, 2.17–2.36) [7,24]. While case fatality rates for white and black patients are similar, race appears to be associated with increased incidence and severity of sepsis, as non-white patients are more likely than white patients to acquire sepsis with a mean annual relative risk of 1.90 (95% confidence interval, 1.81–2.00), and black patients are more likely than white patients to have severe sepsis with a poverty-adjusted incidence rate ratio of 1.44 (95% confidence interval, 1.42–1.46) [23,25,26,27]. While multiple factors may be contributing to these differences such as the effect of estrogen on immune cell function [28], comorbid health factors that are often present in older individuals [24], and racial disparities in healthcare [26,27], recent insight into the role of the microbiome may provide a unifying explanation for these associations (Figure 1).

Changes in the microbiome have been associated with a variety of non-malignant and malignant disease processes [29,30,31]. For hospitalized patients with sepsis, their microbiota changes may be secondary to the multitude of therapeutic interventions to which they are exposed. The administration of antibiotics, analgesics, and anesthetics have all been shown to impact the microbiota diversity and abundance with potentially deleterious effects that may play a role in post-sepsis recovery [32,33,34]. The most well-known clinical example is antibiotic-induced pseudomembranous colitis caused by *Clostridium difficile*. In this circumstance, broad spectrum antibiotics allow for an increased abundance of *C. difficile* in the intestine with resultant inflammation and colitis. However, more subtle changes occur that have long-term consequences. Recent studies have shown that decreased microbiota diversity with increased abundance of the genus *Enterococcus* is observed in critically ill patients and is associated with a higher risk of sepsis, which may give credence to this line of investigation [35,36,37,38,39]. In a study involving 24 long-stay ICU patients, approximately two-thirds of patients were observed to have a loss of microbial diversity based on 16S rRNA gene sequencing of bacteria in the stool. Furthermore, three-quarters of patients were observed to have increased abundance of pathogens *Enterococcus faecium, Klebsiella pneumoniae, Enterobacter cloacae, and E. coli* [38]. More specific to sepsis, a study on stool samples of 103 sepsis patients compared to matched controls on intensive care and hematology units were observed to have a higher abundance of *Enterococcus* based on 23S rRNA gene sequencing (to calculate total abundance) as well [39]. The differential overexpression of this taxa supports the hypothesis that components of the host microbiota can mediate or elevate sepsis risk. Additionally, mortality was noted to be higher in sepsis patients with lower abundance of butyrate-producing bacteria, which is a short-chain fatty acid (SCFA) known to have immunomodulating and protective effects from the intestinal microbiota [40]. Whether these changes are associative or play a more critical role in sepsis and patient outcomes is currently unknown.

### 2.2. Progressing beyond Associations

In order to scientifically and clinically advance beyond these described associative changes, a more mechanistic determination is required. A common component of the diseases that have demonstrated similar associative changes with the microbiome is the pro-inflammatory nature of each of them, which provides a unique entry point to investigate the microbiome as a causative factor of sepsis and post-sepsis recovery. One of the most well-investigated interactions of the microbiome with inflammation is related to inflammatory bowel disease [41]. It is well-documented that intestinal mucosal barrier function plays a key role in preventing intestinal microbiota-driven inflammation through a variety of mechanisms such as microRNA, metabolites, and various TLR and Nod-like receptor (NLR) signaling pathways [41]. The microbial-mediated epithelial and endothelial barrier dysfunction that contributes to sepsis and sepsis-related outcomes is currently a robust area of investigation. The epithelial layer of the gastrointestinal (GI) tract plays a critical role in separating luminal toxins, metabolites, and bacteria from the underlying tissue. This is both in a physical capacity, through cell–cell interactions mediated by tight junctions and adhesins, as well as immunosurveillance capacity by tissue-resident polymorphonuclear leukocytes. Typically, in a well-regulated system, the disruption of either of these components allows bacterial translocation and activates a cascade of deleterious inflammatory processes resulting in the host septic response. Given that even elective surgical cases can result in bacterial translocation [42,43], it demonstrates that any stress to the host can create an environment whereby the host intestinal microbiota can facilitate a state of sepsis. The most well-known direct effects of bacteria on intestinal epithelial barrier function involve pathogenic bacteria that disrupt epithelial barrier function through the production of toxins such as Shiga toxin (enterohemorrhagic *Escherichia coli*), enterotoxin (*Clostridium perfringens*), *Cholerae* toxin, Zonula occludens toxin, and hemmagglutinins (*Vibrio cholera*) or through direct cell adhesion, as seen with enteropathogenic *E. coli*. The mechanisms of action for barrier disruption are unique to each individual toxin but revolve around degradation or the redistribution/internalization of tight junction proteins, which result in bacterial translocation, electrolyte and fluid extrusion, and inflammation. While these are communicable infections involving specific bacteria-mediated barrier disruption, they are unlikely to be seen in patients who develop sepsis. However, it demonstrates the capacity of bacteria to impact intestinal barrier function and the systemic response.

It is known that bacterial activation of the inflammasome leads to systemic inflammatory activation. As part of the innate immune system, to monitor and take action against potentially harmful stimuli, inflammasomes act as intermediaries to propagate inflammatory effector signals. The inflammasome process consists of a stimulatory signal, inflammasome “assembly”, and propagation of effector signals [44]. While host-derived signals (DAMPs) can activate inflammasome assembly, of interest in the microbiome-sepsis axis are PAMPs. These signals can be direct pathogen interaction or microbial-derived products and activate the *canonical* inflammasome (as opposed to the *non-canonical* pathway, which is activated by host factors, reactive oxygen species, and mitochondrial dysfunction). After intracellular translocation, the signals activate the NLRC4 or NLRP3/6/7 sensors, which ultimately result in pro-caspase 1 and the activation of IL-1β and IL-18, which leads to downstream inflammation. Much research has reported on the inflammasome and activation by more pathogenic bacteria such as *Listeria monocytogenes* and enterohemorrhagic *Escherichia coli* [45], but these are not necessarily germane to in-hospital sepsis. However, NLRP3 can be activated by common culprit Gram-positive and Gram-negative bacteria found in septic patients such as *Staphylococcus aureus* and *Streptococcus pneumoniae* [46,47,48]. Interestingly, polymorphisms of the NLPR3 gene have been demonstrated to impart gain-of-function that resulted in a suppression of NLRP3 expression and downstream inflammatory activation, which resulted in protection of patients from progression of sepsis [49].

## 3. Manipulation of the Microbiome for Septic Patients

**Figure 2:**
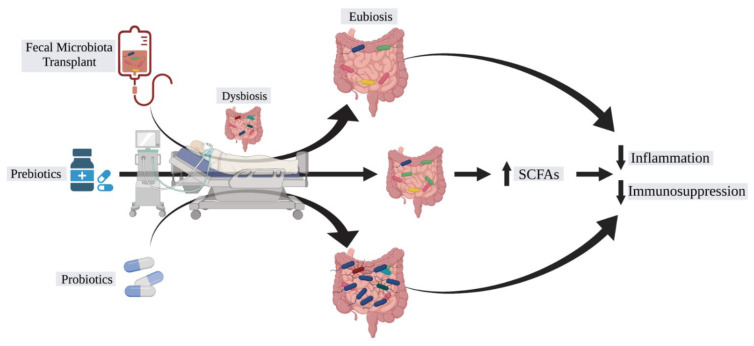
Therapeutic potential of the microbiome in sepsis. Currently tractable systems of microbiome-mediated treatment of sepsis. Fecal microbiota transplantation (FMT) serves to reconstitute the patient’s intestinal microbiota with the microbiota of a healthy donor to restore eubiosis and normal metabolic function of the microbiota. Alternatively, prebiotics introduce products into the gut that the existing microbiota can utilize for beneficial purposes for the host, such as the introduction of dietary fiber to increase the abundance of short-chain fatty acid (SCFA) fermenting microbes that yield increased butyrate and propionate, for example. Finally, probiotics introduce specific “beneficial” bacteria to the existing microbiota in an effort to outcompete the deleterious microbes. The end goal is to decrease inflammation and immunosuppression such that the process of sepsis can be subdued.

With growing interest in the role of the microbiome in disease pathogenesis, interest in a “magic bullet” to manipulate the microbiome has grown as well (Figure 2). The potential for antibiotic alteration of the microbiome for disease control has shown some promise in Crohn’s disease, but in general, this non-targeted approach has limited applicability for the majority of diseases [50]. While this approach as well as bacteriophage therapy focuses on the *elimination* of culprit microbiota, [51] a more practical approach may be *restoration* of a healthy microbiota. Such restoration would be in the form of probiotics or fecal microbiota transplantation (FMT). The use of FMT has shown limited success in recalcitrant *Clostridium difficile* infection [52], but prior FMT studies have also demonstrated fatalities secondary to the transmission of antibiotic-resistant bacteria which should bring pause to uniform application of this treatment strategy to other infectious diseases, such as sepsis [53]. Nevertheless, the therapeutic potential is intriguing and has not prevented clinical trials from being undertaken in patients with sepsis and sepsis prevention (Table 1) [54]. Fecal transplantation in the management of critically ill patients is still in the early stages of research with case reports showing evidence of sepsis cure [55,56]. Probiotics involve the administration of “beneficial” bacteria such as Lactobacillus and Bifidobacterium, but what constitutes a “beneficial” microbe may be determined as much by the microbe as the recipient [57]. A randomized controlled trial in 2016 showed promising data that probiotic treatment in critically ill patients is associated with decreased risk of infections including ventilator-associated pneumonia [58]. However, probiotic use has come into question due to recent studies uncovering the potential adverse effects of probiotic use in ICU patients such as Lactobacillus bacteremia [59]. In order to circumvent the potential detrimental effect of FMT or direct probiotic introduction, prebiotics aim to introduce products into the gastrointestinal tract that serve to induce the growth of healthy microbes. Most notable is the introduction of dietary fiber to induce the propagation of SCFA-producing bacteria, which has protective immunomodulating effects as described previously. These serve as the intermediary between diet and direct metabolic effect.

As advancements in sequencing technology and computational tools have improved, our ability to analyze data on the human microbiome and a deeper knowledge of the metabolic impact of the host microbiota through metabolomics will potentially allow targeted therapeutics to alter sepsis risk and outcomes [60,61,62]. A mouse study of pneumonia-derived sepsis has demonstrated not only alterations of the microbiota in septic mice, but through metabolomic analysis, identified the importance SCFAs with decreased levels of acetate, propionate, and butyrate [63]. While the results of this study should not be overstated, given its associative nature, therapeutic replenishment of such SCFAs would be needed to prove causation.

## 4. Conclusions

While advances in critical care medicine have resulted in improved sepsis survival, disparities continue to render certain individuals at higher risk for sepsis and sepsis-induced mortality. The microbiome has emerged as a major player in the pathogenesis of sepsis. Evidence is accumulating for the human microbiome to not only be associated with the development of sepsis and its complications but also contribute to ongoing inflammatory pathways involved in the sepsis process. Through a deeper understanding of the microbiome–sepsis interplay, therapeutic targets can be identified to enable clinicians to take advantage of the microbiome in the management of sepsis. At present, attention should be placed on clinical trials that aim to stabilize dysbiotic microbiota caused by sepsis or that supplement beneficial bacteria or their metabolic by-products. Finally, as was the case for the microbiome and cancer, the majority of investigations involving the microbiome and sepsis center around bacteria and the gut microbiota. Little evidence exists regarding the role of virome, fungome, and other microbial metagenomes as well as organ-specific sites of sepsis (kidney, lungs, liver). Much research, but much excitement, lies ahead for this ever-expanding and needed field of investigation.

## Figures and Tables

**Table 1:** Overview of clinical trials investigating the role of the microbiome in the care of sepsis patients.

Study	Objective	Cohort	Intervention	Location	Status
Gut Microbiome Dysbiosis in Sepsis-Induced Coagulopathy	Analyze gut microbiome alternations and coagulation studies in ICU adult patients	ICU adult patients	Observational	China	Recruiting
Predicting EONS in PPROM Patients (PEONS)	Analyze microbiome via 16S rRNA sequencing of neonates with and without early-onset neonatal sepsis	Neonates with and without early-onset neonatal sepsis	Observational	Germany	Active, not recruiting
Novel Mechanisms and Approaches to Treat Neonatal Sepsis	Characterize immune genomic expression and microbiome in preterm and term neonates	Preterm and term neonates, healthy adult controls	Observational	United States	Recruiting
Molecular Diagnosis and Risk Stratification of Sepsis in India (MARS-India)	Characterize immunoinflammatory status and microbiome in septic patients	Septic patients, non-septic ICU patients, healthy controls	Observational	India	Recruiting
Study of Early Enteral Dextrose in Sepsis (SEEDS)	Study how early enteral dextrose infusion in septic patients impacts serum pro-inflammatory IL-6 levels	Septic patients	Enteral dextrose infusion vs. enteral water control	United States	Completed
Characterization of Intestinal Microbiota Stability in Preterm Born Neonates (NEC)	Analyze gastrointestinal microbiome in preterm infants for association with risk of developing NEC/LOS	Preterm infants with and without NEC/LOS	Observational	Switzerland	Recruiting
SEPSIS Observational Cohort Study in Young Infants in Bangladesh	Analyze gastrointestinal microbiome in young infants for association with risk of developing severe infection	Young infants	Observational	Bangladesh	Recruiting
Prebiotic Fiber to Prevent Pathogen Colonization in the ICU	Study how fiber supplementation in ICU patients impacts pathogen colonization/infection	ICU adult patients	High fiber diet vs. lower fiber diet	United States	Completed
Effect of Gut Microbiota on the Prognosis of Sepsis	Analyze relationship between gut microbiota and prognosis of sepsis	Adult patients with sepsis	Observational	China	Not yet recruiting
The Role of the Microbiota in the Systemic Immune Response (MISSION-1)	Study how depleting gut microbiota impacts systemic immune response	Healthy adults treated with antibiotics	Antibiotics (ciprofloxacin, vancomycin, metronidazole)	Netherlands	Completed
Human Milk Fortification in Extremely Preterm Infants (N-forte)	Study how bovine-milk based fortifier in extremely premature infants impacts incidence of NEC, culture-proven sepsis, and mortality	Extremely premature infants	Bovine milk-based fortifier vs. control fortifier	Sweden	Recruiting
Bovine Colostrum as a Human Milk Fortifier for Preterm Infants (FortiColos-Ⅱ)	Study how bovine colostrum in preterm infants impacts weight gain, NEC incidence, and late-onset sepsis incidence	Preterm infants	Bovine Colostrum fortifier vs. control fortifier	China	Recruiting
Oropharyngeal Administration of Mother’s Colostrum for Premature Infants	Study how mother’s colostrum in extremely premature infants impacts incidence of late-onset sepsis, NEC, and VAP	Extremely premature infants	Oropharyngeal mother’s milk vs. oropharyngeal water control	United States	Active, not recruiting
Bovine Colostrum as a Fortifier Added to Human Milk for Preterm Infants (FortiColos)	Study how bovine colostrum in preterm infants impacts weight gain, NEC incidence, and late-onset sepsis incidence	Preterm infants	Bovine Colostrum fortifier vs. control fortifier	Denmark	Recruiting

## Data Availability

Not applicable.
